# How Does Office Design Support Employees’ Health? A Case Study on the Relationships among Employees’ Perceptions of the Office Environment, Their Sense of Coherence and Office Design

**DOI:** 10.3390/ijerph182312779

**Published:** 2021-12-03

**Authors:** Melina Forooraghi, Elke Miedema, Nina Ryd, Holger Wallbaum

**Affiliations:** Department of Architecture and Civil Engineering, Chalmers University of Technology, 412 96 Gothenburg, Sweden; elke.miedema@chalmers.se (E.M.); nina.ryd@chalmers.se (N.R.); holger.wallbaum@chalmers.se (H.W.)

**Keywords:** office design, flexible office, health, salutogenic, sense of coherence, well-being, workplace design, case study

## Abstract

This study investigated the current design circumstances of an office as well as employees’ perceptions of the office environment in relation to their perceived health, drawing on sense of coherence theory (comprehensibility, manageability, and meaningfulness). Previous studies have related the physical office environment to employee health. However, most studies have focused on alleviating negative effects, while health-promoting potential, including employee sense of coherence, has been overlooked. This study adopted a mixed method case study approach, combining semi-structured interviews with employees, structured observations, and analysis of architectural drawings. The results indicated that employees’ perceptions did not always align with the ideas behind the architectural design and that employees understood the environment differently. The study also highlighted the interrelations (and contradictions) among the different components of sense of coherence. The findings imply that organizations may need to prioritize which components of coherence should be supported most by the office environment. It also suggests that case-specific design aspects should play a more central role in studying and conceptualizing healthy office design and that design solutions should be continuously modified during the use phase, while ensuring employees’ participation. The study concluded that an ‘ideal’ office environment should not be the goal. Instead, office design should provide an environment in which employees are able to cope with challenges in comprehensible, manageable and meaningful ways.

## 1. Introduction

How can we plan, design, and manage healthy office environments? Studies show that the physical office environment influences employees’ health. However, most studies have focused on identifying risk factors for health (pathogenic aspects) [1,2,3,4]. Meanwhile, the health-promoting potential (salutogenic aspects) of office environments, such as nature references as a means of recovering from stress or space personalization as a means of enhancing well-being, have often been overlooked [1]. The salutogenic concept ‘sense of coherence’ (SOC) explains how people manage to stay healthy in stressful situations. SOC is the ability of a person, a community or a society to overcome challenges by (i) understanding the character of the problems (comprehensibility), (ii) identifying and deploying relevant resources (manageability), and (iii) viewing the perceived problems as meaningful challenges and worthy of engagement (meaningfulness) [5]. The components of SOC are resources that may protect individuals from stress and reduce health risks [6,7]. Examples of such resources are education, material resources, coping strategies and social support [8]. People with a higher SOC adopt healthier behavior and are more motivated to cope with stressors and thereby become more resilient with better perceived health and quality of life [7,9,10,11]. While organizations are increasingly implementing flexible office concepts, we know little about which architectural design features in such environments improve employees’ SOC. Hence, understanding SOC in the context of flexible office environments will contribute to beneficial health outcomes.

Flexible office concepts, such as activity-based and combi offices, are intended to support flexible ways of working. In these office concepts, workstations are combined with back-up spaces, such as quiet rooms for concentrated work, phone booths for calls, meeting spaces for collaborations, to support work activities [12]. The main difference between the two abovementioned office concepts is desk ownership. In combi offices, a desk is assigned to each employee, while in activity-based offices, desks are shared among employees [13]. Having assigned workstations in addition to access to back-up spaces results in a different office experience in combi offices than in activity-based offices, as well as in added floor area per employee and eventually higher costs. Hence, there is great value in addressing combi offices more in depth to understand the ways in which they influence employee SOC.

Studies suggest that architectural design features such as lighting, layout, furniture, acoustics, privacy and the process of relocation/modifications have an impact on how employees experience their new offices [14,15,16,17,18]. However, these case-specific features have rarely been addressed in the literature from a design perspective [19,20]. Brunia et al. [14] suggested that the main differences between the best and worst flexible offices concern employee satisfaction with the interior design, level of openness, subdivision of space, number and diversity of workspaces and accessibility of the building, as well as the implementation process. Hence, if these features are essential in understanding employees’ experience of office environments, then this gap in the literature points to whether research findings from different cases are comparable, especially when these aspects are not considered in the studies. In addition, studies have indicated that flexible offices are often used differently than intended. For instance, employees do not switch places as often as intended in activity-based offices [14,21,22]. Canter, a human geographer, argued in the book The Psychology of Place that people’s behavior in different places may derive from their previous experiences and their conception of the place. Thus, to fully understand people’s responses to places and their reactions within them, we need to understand what and how they think [23]. This points to a need for a more in-depth understanding of how employees appropriate their office environment.

Given that the case-specific design aspects play a key role in how employees experience office environments, qualitative and in-depth research approaches appear particularly relevant to (i) further understanding of architectural design features that are important to employee SOC; (ii) creating a more accurate evidence base for comparison; and (iii) planning, designing and maintaining healthy office environments. In addition, qualitative studies on the effects of office use on employee health are scarce [24]. Based on these identified gaps in the literature, we see a need to integrate a design perspective into office studies. The overall goal of the study is to contribute to the understanding of interrelations between the employees’ perceptions of office environment, their SOC and office design. The research questions are as follows:

RQ1: What are the current office design circumstances?
RQ2: In what way(s) do employees’ perceptions of the office environment relate to their SOC?

## 2. Theoretical Framework

This paper adopts the conceptualization of health proposed by Huber et al. [25]: ‘the ability to adapt and to self-manage in the face of social, physical and emotional challenges’. This conceptualization fits with the salutogenic approach in which health is viewed as a dynamic concept on a health-ease and dis-ease spectrum. Salutogenesis was introduced by Antonovsky [26], who argued that conventional health approaches focused on the treatment of disease (pathogenic) while neglecting the factors that create health (i.e., salutogenic approach) [26]. Later, he argued that health ultimately depends on the individual’s ability to create and maintain the SOC [5].

A resourceful (physical) working environment helps employees build up a SOC which leads to greater work engagement [27]. However, SOC in the built environment is mostly addressed in relation to healthcare building design. For instance, the design of the waiting room influences the number of people who can be seen in one space at the same time, influencing a sense of crowding and thereby decreasing manageability [28]. In the office context, few studies have applied salutogenesis. For instance, Ruohomäki et al. [29] adopted a salutogenic approach toward office relocation at a conceptual level, but no explicit relation was made to SOC. Roskams and Haynes [30] proposed a conceptual framework that identified environmental demands and resources influencing SOC, such as a clear set of behavioral rules, biophilic design solutions, and design that supports social cohesion, physical activity, and personal identity expression. Similarly, a recent longitudinal case study investigated indicators of SOC during relocation to an activity-based office with a questionnaire and focus group interviews [31]. The study showed that all SOC indicators were positively associated with overall health, well-being, and work satisfaction. Meaningfulness, manageability and comprehensibility significantly increased from baseline to nine months post-relocation; the implementation process facilitated SOC with support, tools on how to work in an activity-based office, communication and preparatory activities pre-relocation.

**Figure 1:**
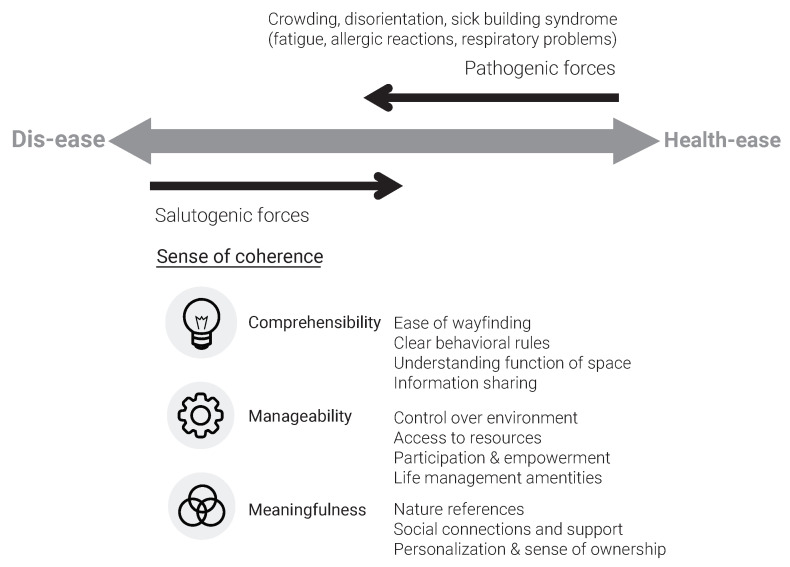
Sense of coherence in an office environment, adapted from Forooraghi et al. [32].

This paper builds upon Forooraghi et al. [32], who applied SOC theory in a longitudinal study and proposed several architectural features per component of SOC (Figure 1).

*Comprehensibility* in the office context relates to the features that make the environment structured, predictable, and explicable.

**Wayfinding** refers to attributes of the environment facilitating or hindering easy orientation.
**Understanding the function of the space** refers to attributes of the environment that communicate the function of a space.
**Behavioral rules** refer to attributes of the environment and/or agreements between employees and organizations indicating expected behavior in the physical environment.
**Information sharing** refers to attributes of the environment as well as organizational procedures in which practical information about relocation and/or office maintenance is clearly communicated.
*Manageability* in the office environment relates to the features that enable access and control over resources needed to cope with the challenges of the environment.
**Control over the environment** refers to attributes of the office facilitating or hindering control over perceived stimuli.
**Access to resources** refers to attributes of the environment facilitating or hindering access to preferred/needed technical equipment, furniture, and storage space.
**Participation and involvemen**t refer to change processes facilitating or hindering building user involvement.
**Life management** refers to amenities that facilitate or hinder employees in balancing the pressure of work life.
*Meaningfulness* in the office environment relates to features that evoke meanings in the environment.
**Nature references** refer to attributes that enable access to the elements of the natural environment.
**Social connections and support** refer to attributes of the environment facilitating or hindering social interactions and feelings of community.
**Personalization and sense of ownership** refer to attributes of the environment facilitating or hindering building users asserting meaning to space through identity expression.

## 3. Method

**Figure 2:**
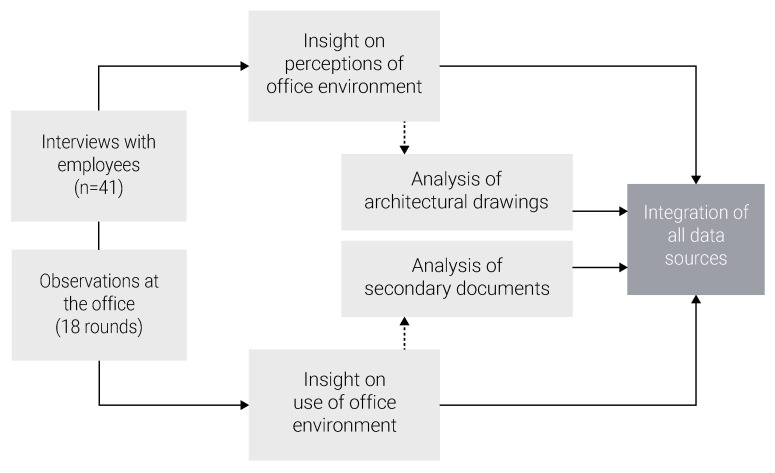
Research design.

A mixed-method case study approach was adopted to investigate the current office design circumstances as well as employees’ perceptions of the office environment in relation to their SOC. Data collection involved semi-structured interviews with employees, structured observations, architectural drawings, and secondary documentations. This methodology followed a convergent-parallel design; i.e., data were collected by different methods in parallel, were analyzed separately, and then the findings were compared, contrasted and integrated [33], as displayed in Figure 2.

The qualitative research approach was chosen for an in-depth study of the subjective nature of individuals’ SOC [33]. The qualitative data from the interviews were used to gain a deeper understanding of employees’ insights and experiences on how comprehensible, manageable, and meaningful their office environment was.

### 3.1. Case Context

The case concerns a university department that relocated from cell offices into a renovated combi office in August 2017. The relocation was combined with an organizational merger bringing together 10 different divisions of employees into one department. Three out of ten divisions had their offices in the same building prior to renovation, and the rest came from other buildings on the campus.

**Figure 3:**
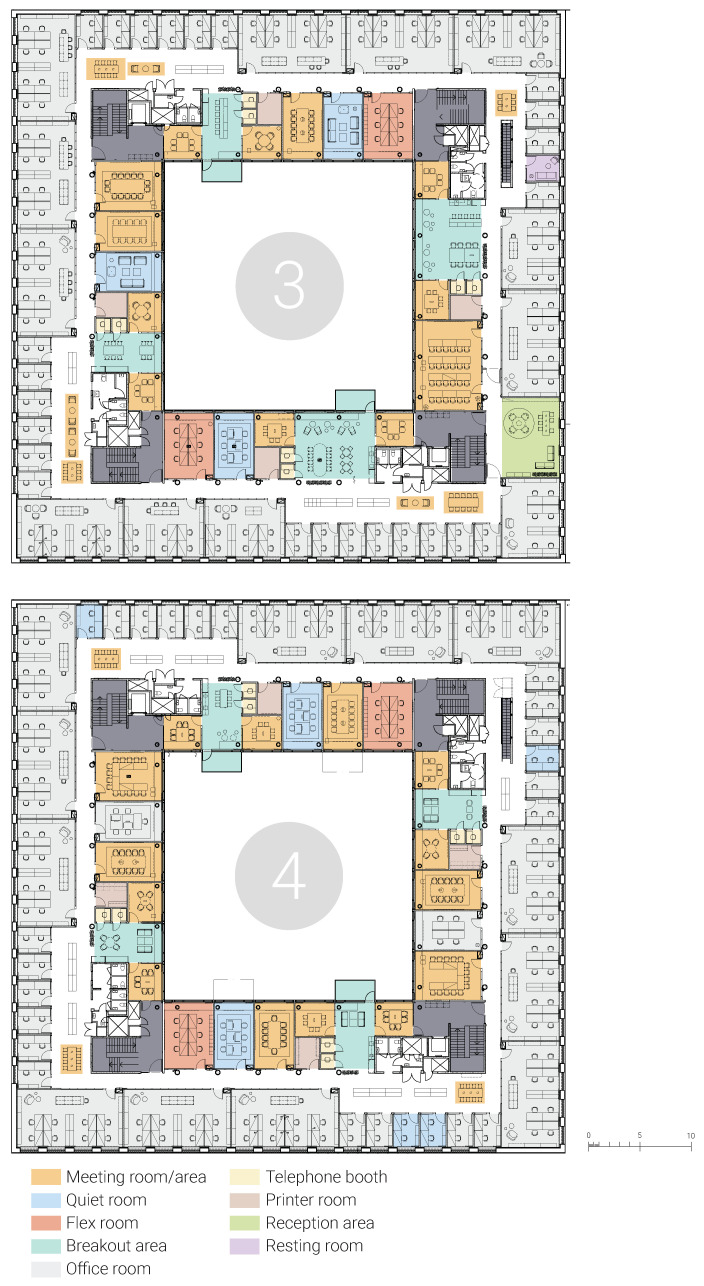
Floor plans of the studied office.

The study focuses on the staff areas on the third, fourth (offices and back-up spaces) and fifth (only staff lunchrooms and meeting spaces) floors (Figure 3). The building was situated around an atrium, with a central corridor system. The spaces adjacent to the atrium were back-up spaces, including meeting rooms, phone booths, quiet rooms, flexible room/offices, breakout areas and balconies. The spaces on the outer façade included mainly offices (two, six or eight employees) and a reception area. The employees were assigned to an office room or specified desk group per division. All staff areas, including all offices, were accessible to all staff with keycards. Students did not have access to these staff areas, except for a few meeting rooms on the fifth floor.

The office interior was modified two years post-relocation by the facility management: (i) A quiet room with couches was turned into a shared office room due to a lack of workstations, (ii) a windowless meeting room was turned into a printing room, following complaints about lack of printers, (iii) translucent curtains were added to office rooms facing the staircases to enhance visual seclusion, and (iv) couches in the lunchroom were moved to other breakout areas on fourth floor and replaced with dining tables and chairs.

### 3.2. Study Population

All university employees of the department were invited via email and posters on site to participate in the study. Forty-one employees volunteered to participate in the interviews (Table 1). The participants had different roles and responsibilities as well as durations of time working at the university. They were considered good informants, meaning they were able and willing to contribute to the study.

### 3.3. Data Collection Procedure

Data were collected in September 2019, two years post-relocation, through individual semi-structured interviews, structured observations, and review of architectural drawings and secondary documents, such as a building guidebook.

The interviews averaged an hour, and they were audio recorded. The questions were designed to enable the interviewees to share their insights on how they experienced the office environment, their activities and preferences (Table 2). A card-sorting exercise as well as floorplan drawings, markers and notes were used as mediation tools during the interviews [34,35]. The card sorting exercise consisted of a biaxial chart visualizing levels of satisfaction and importance and a set of cards relating to predefined themes to be sorted on the chart. The themes covered office environment features, such as behavioral rules, personal storage, acoustic and visual privacy. The participants were asked to sort the cards one by one while describing the motivation for their choice. At the end of the exercise, blank cards were also given, in case the participants wanted to raise new topics for discussion. The drawings, markers and notes aided interviewees to elaborate on their explanations, describe their routines and space use, or signal relevant aspects of these spaces.

The observations in the office were structured observations; i.e., a systematic plan with a predefined route was used, and employees were aware of the observer. A total of 18 rounds were conducted by the first author. Each predefined route included walking around all the workstations, back-up spaces and breakout areas, with the observer taking structured field notes and drawing annotations as well as pictures. The field notes indicated, for example, the workstations and back-up spaces in use, the number of employees per space, the available facilities and equipment, flows of people between spaces, and whether different spaces were organized and orderly. The rounds were scheduled according to the availability of the observer, avoiding events that were not part of the daily routine of employees and caused abnormal occupancy rates, such as a monthly department meeting. The observations were conducted over two weeks and across four intervals (8:00–10:00, 10:00–12:00, 13:00–15:00, and 15:00–17:00), with the aim to cover the equivalent of a regular Monday to Friday working week.

Secondary documentation included an office in-house guide with plans and behavioral guidelines. Such documentation was collected from the university’s internal website, which is accessible to all employees. The architectural drawings, i.e., floor plans, were provided by the facility management and were updated by the first author to be used for observation rounds and further analysis.

### 3.4. Data Analysis Procedure

The data analysis consisted of multiple iterative stages, including content analysis of the interviews, descriptive analysis of the observation rounds, and floor plan analysis of the building material.

The interviews were transcribed and coded using NVivo 12. An abductive approach was adopted to analyze the content; combing an inductive and deductive approach, defined as ‘creative inferential process’. That is using empirical data and theoretical prepositions in a dialogical process for analyzing qualitative data [36]. The first step involved analyzing the interview transcripts to identify recurring themes related to perceptions of the office environment (see examples in Table 3). This allowed to identify positive and negative perceptions of office environment features. For instance, 25 interviewees referred to ‘exposure to visual stimuli’, which in step 2 was coded under ‘control over the environment’. In a further deductive round of coding (step 3), the office environment features were related to the components of SOC: comprehensibility, manageability and meaningfulness. The first two authors coded the transcribed interviews separately. Whilst consistency was high, any differences were discussed, and resolved by discussion. Furthermore, the four authors regularly discussed the analysis, data triangulation and reporting strategy during the process.

Furthermore, data from the observations were analyzed to support and complement the findings from the interviews. This involved reviewing and summarizing observation field notes and occupancy data. Occupancy was calculated for office rooms based on the percentage of workstations occupied with respect to the maximum number of workstations. Utilization was calculated for back-up spaces based on the percentage of the total number of 18 observation rounds that the spaces were observed in use.

The architectural drawings and secondary documents were analyzed from a design perspective to support and complement the findings. That is, the employees’ perceptions were contrasted with observation data, architectural drawings, secondary data, and pictures of the office to understand the underlying reasons for these perceptions. For instance, exposure to visual stimuli was mentioned as a negative feature by the majority. The level of transparency observed in drawings and observations confirmed that the extensive use of glass partitions led to a high level of exposure to visual stimuli. Another example is that when interviewees referred to behavioral rules, the in-house book was analyzed to determine what type of information the organization had communicated about expected behavior in shared office rooms and back-up spaces.

The triangulation of multiple data sources followed a parallel convergent design, in which the data from different data sources were analyzed independently and brought together during the interpretation [33].

Approval was obtained from the head of the department to carry out the study. Prior to being interviewed, all participants were informed verbally and in writing about the purpose of the study, that their participation was voluntary, that they could end their participation at any time, and that they could choose not to answer any questions. They were also informed that the personal information would be known only by the research team and would be protected according to the General Data Protection Regulation. Informed consent was obtained when the participants agreed to complete the survey.

## 4. Findings

The findings are presented in three sections that reflect the SOC components (comprehensibility, manageability and meaningfulness) and their subthemes, according to the SOC framework by Forooraghi et al. [32]. All sections describe the current office design setting based upon on-site observations and analysis of architectural drawings as well as secondary documentation. Then, employees’ perceptions of the respective features are described. Each colored bar illustrates one to seven interviewees who reported the (positive or negative) office environment perceptions.

### 4.1. Comprehensibility

**Figure 4:**
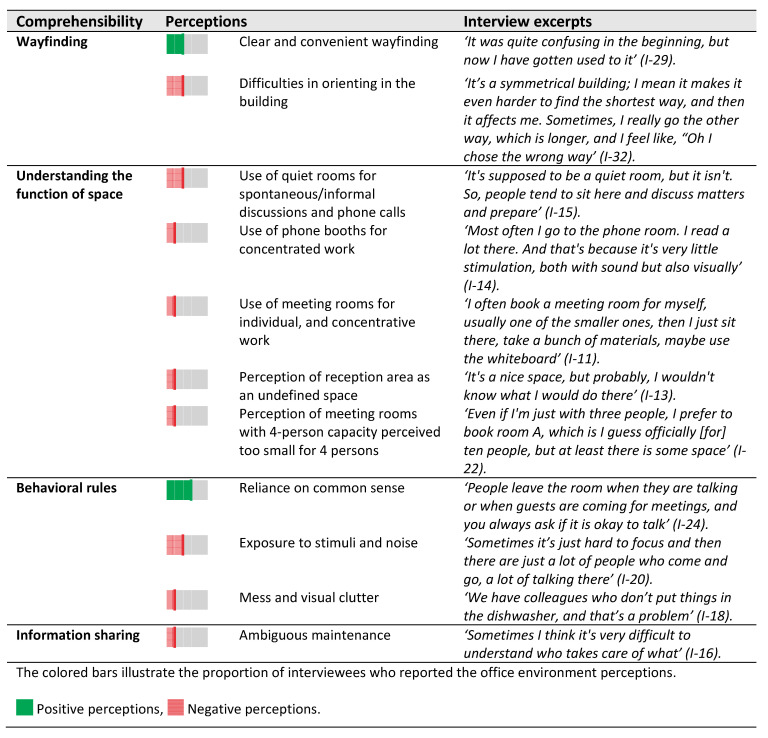
Perceptions of comprehensibility in the office environment as reported in interviews.

The perceptions of the office environment show that comprehensibility was associated with wayfinding, understanding the function of the space, and behavioral rules, as presented in (Figure 4).

#### 4.1.1. Wayfinding

As mentioned, wayfinding refers to attributes of the environment facilitating or hindering easy orientation.

**Figure 5:**
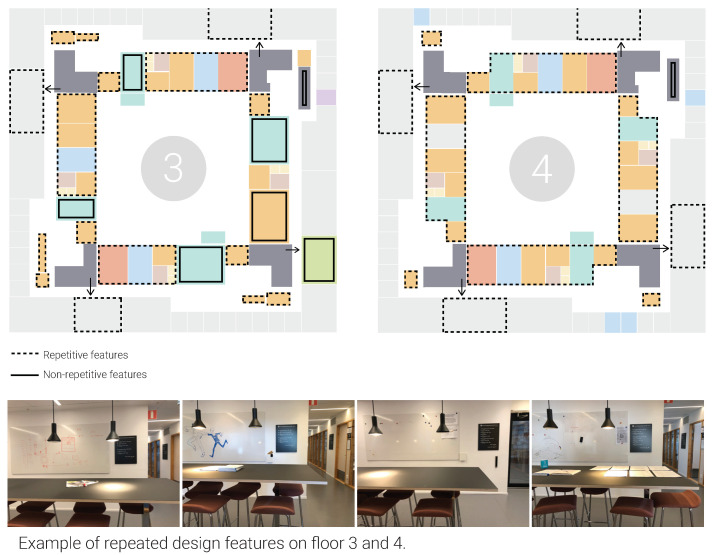
Wayfinding in the office space.

Design setting: The observation and floor plans show the symmetrical layout, and the deficiency of distinct design features, i.e., landmarks, in addition to the use of identical and repetitive furniture and colors in all corridors and corners, could lead to navigation challenges, especially for those who were not familiar with the building (Figure 5). That said, building users could find their way after making a few laps due to the square layout of the building.

Perceptions: The interviews indicated that wayfinding had varied influences on how employees comprehended the layout of the office. One-third of the interviewees (13/41) reported that the orientation in the building became intuitive after becoming used to the labeling system and the layout. Nevertheless, others (12/41) found wayfinding difficult due to the monotonous look and the square layout (Figure 4).

#### 4.1.2. Understanding the Function of the Space

Understanding the function of the environment refers to the attributes of the environment that communicate the function of the space.

**Figure 6:**
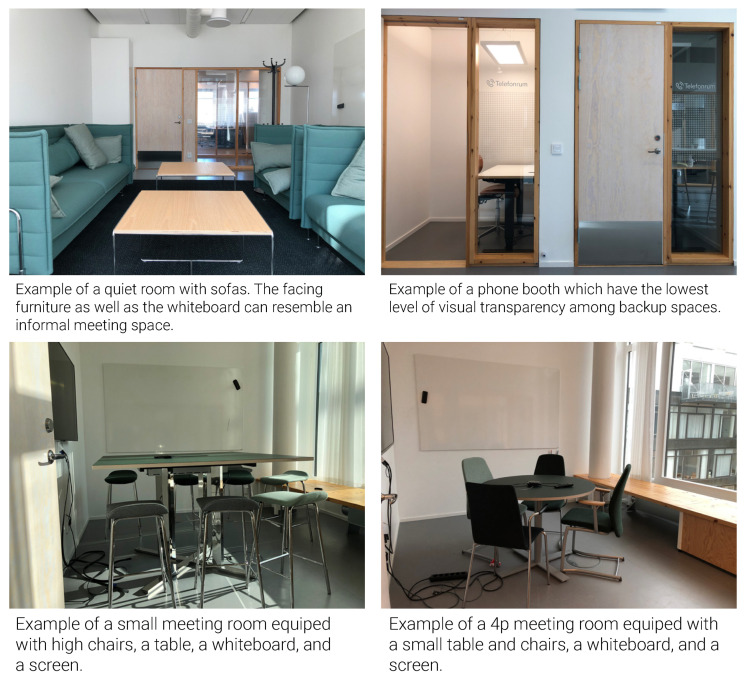
Pictures illustrating interviewees’ understandings of the function of spaces.

Design setting: Most spaces had signs outside and inside of the door indicating the room number and its intended use. Exceptions were the phone booths, which had signage/labels on the window (Figure 6). The ability to understand the function of the environment could be influenced by the furniture setup and spatial characteristics of the spaces. For instance, phone booths were spatially secluded and thus had minimum visual and acoustic distractions. The quiet rooms could not be reserved, and they had soft seating (i.e., two armchairs, a sofa, and a pouf) that faced each other and a whiteboard; they thus resembled spaces that are typically used for face-to-face informal meetings. See more examples in Figure 6.

Perceptions: The different functions mentioned by the interviewees included offices, meeting rooms, phone booths, and quiet rooms. The interviewees expressed alternative activities in these spaces. For instance, spaces labeled phone booths were found to be suitable for engaging in concentrated work with minimal distraction and signaling unavailability to colleagues (Figure 4). Quiet rooms with sofas were perceived as suitable spaces for informal discussions. These rooms were however the least often occupied rooms among back-up spaces (Table 4). More examples of alternative uses are presented in F.

#### 4.1.3. Behavioral Rules

Behavioral rules refer to attributes of the environment and/or agreements between employees and organizations indicating expected behavior in the physical environment.

Design setting: An in-house guidebook was given to employees upon their relocation to the new office in August 2017. The guidebook provided information about the new premises and office etiquette. However, the book was not updated after the office modifications were implemented in August 2019. The book was mentioned only once in the interviews. According to the guidebook, employees should comply with the following guidelines:

show consideration and respect
be clean and tidy—leave common areas as you would want to find them
enjoy the shared space but please leave private furniture, textiles and plants at home
maintain peace and quiet—it is important to keep your voice down and avoid talking across the room
when necessary, use a meeting room or other suitable space for lengthy discussions
use headphones when listening to music, the radio and so on
show consideration in the use of perfumes or other scents
if somebody’s behavior disturbs you, do say so—but try to give constructive feedback.

Nevertheless, the expected level of cleanliness and individual responsibilities were not clearly communicated. Instead, employees were asked to keep the common areas ‘as you would want to find them’.

Perceptions: Almost all interviewees reported that they did not have any agreements on how to behave in the office rooms, regardless of the type of office (Figure 4). More than half of the interviewees (22/41) had a positive view of relying on common sense, but others (12/41) felt disturbed by the ambiguity about expected behavior and individual responsibilities concerning cleanliness and order. One-third of the interviewees (14/41) perceived that a lack of behavioral rules made the office environment less comprehensible.

#### 4.1.4. Information Sharing and Transparency

Information sharing refers to attributes of the environment as well as organizational procedures in which practical information about relocation and/or office maintenance is clearly communicated.

Design setting: The phone numbers of maintenance service were provided at the door of every room, providing access to information. However, it was not communicated whether a problem report was already being processed.

Perceptions: The office was perceived as less comprehensible by a minority of interviewees (8/41) due to ambiguous facility management strategies/processes. Some interviewees reported that the maintenance service was unresponsive to problem reports, especially concerning the automated shades.

### 4.2. Manageability

**Figure 7:**
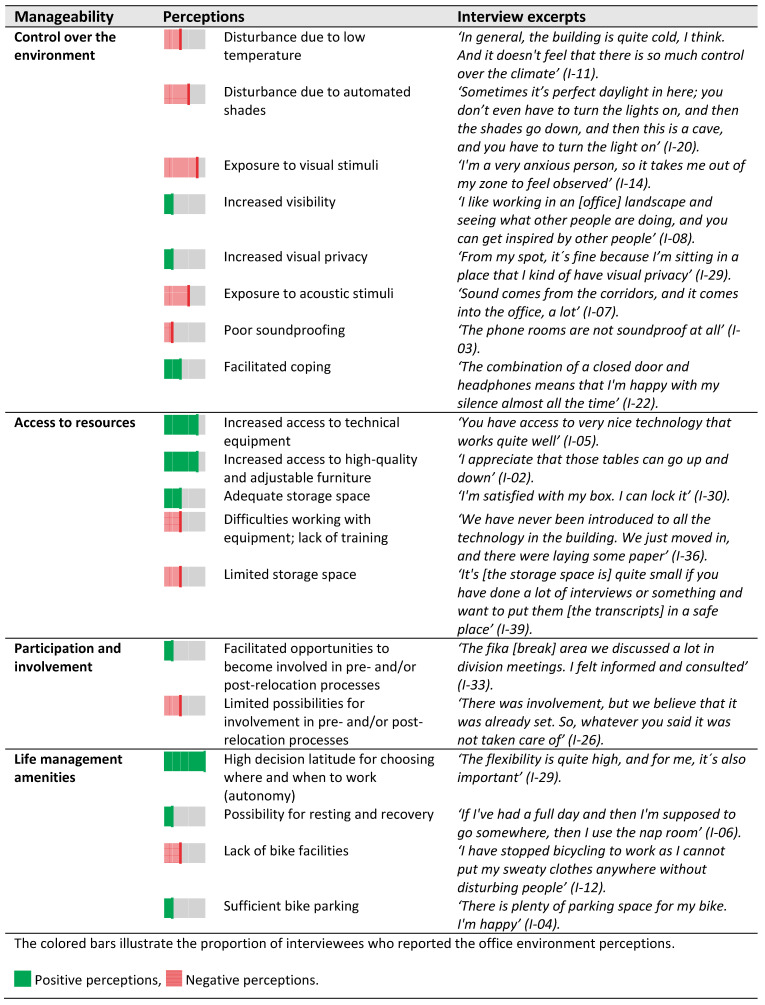
Perceptions of manageability in the office environment as reported in interviews.

The findings show that the office environment influenced manageability through control over the environment, participation and involvement, access to resources and life management amenities, as presented in Figure 7.

#### 4.2.1. Control over the Environment

Control over the environment refers to attributes of the office facilitating or hindering one’s control over perceiving visual and acoustic stimuli.

**Figure 8:**
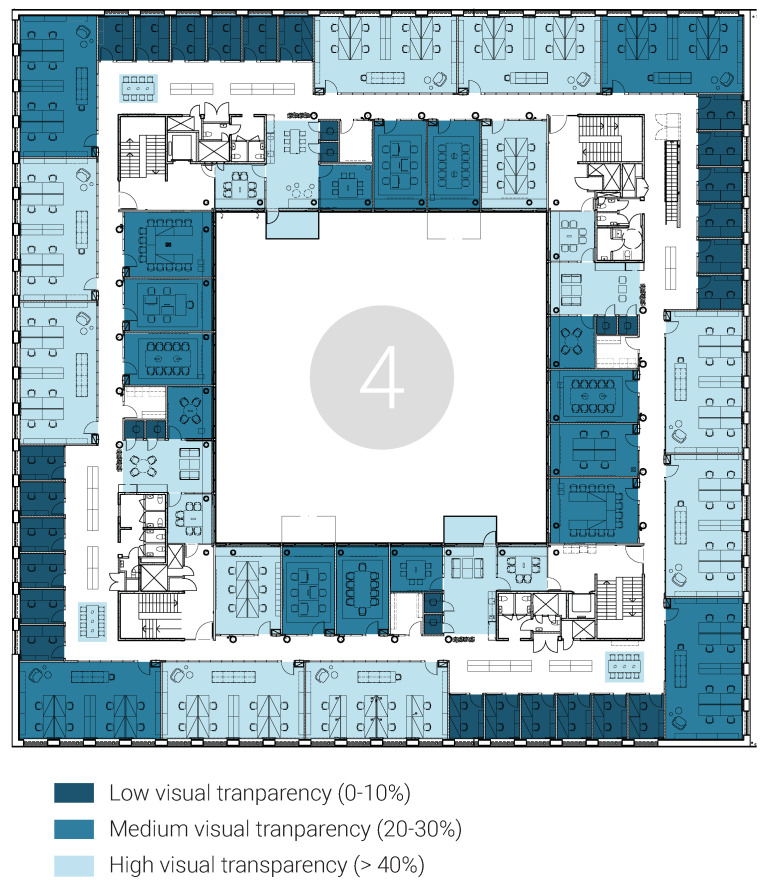
Example of visual transparency on floor 4, calculated as the ratio of the glass/open area to solid wall area.

Design setting: The office offered limited personal control over the environment. The climate system was centrally regulated. Additionally, the shades were entirely automated with a sensor that reacted to the amount of daylight outside, at one floor at the same time, with the consequence that the rooms with less daylight became even darker. The extensive use of glass partitions in all office and back-up spaces increased the level of transparency (Figure 8). That said, some rooms or workstations had a more protected position. For instance, some two-person office rooms were located behind an internal staircase that functioned as a separating shield from the main corridor; in addition, the corner workstations in the eight-person office rooms had a more visually protected position. The noise coming from the corridor could be explained by the spatial arrangement of the meeting rooms opposite the office rooms, where the corridors or printer rooms would function as a meeting point.

Perceptions: In general, most interviewees perceived limited possibilities to control their environment, i.e., temperature, automated shades, and visual and acoustic stimuli, which reduced office manageability. Temperature was regarded as too low, with no possibility of influence by two-thirds of interviewees (28/41). Over half of the interviewees (22/41) were dissatisfied with the automated shades due to them malfunctioning and offering limited access to daylight. A lack of control over visual stimuli was experienced by 25/41 interviewees, and only four interviewees appreciated the increased spatial transparency. One-third of interviewees (14/41) were satisfied with visual privacy, all of whom had almost secluded workstations. A similar pattern was observed for half of the interviewees (20/41) perceiving a lack of control over acoustic stimuli, and thus the office environment was perceived less manageable.

#### 4.2.2. Access to Resources

Access to resources refers to attributes of the environment facilitating or hindering access to preferred/needed technical equipment, furniture, and storage space.

**Figure 9:**
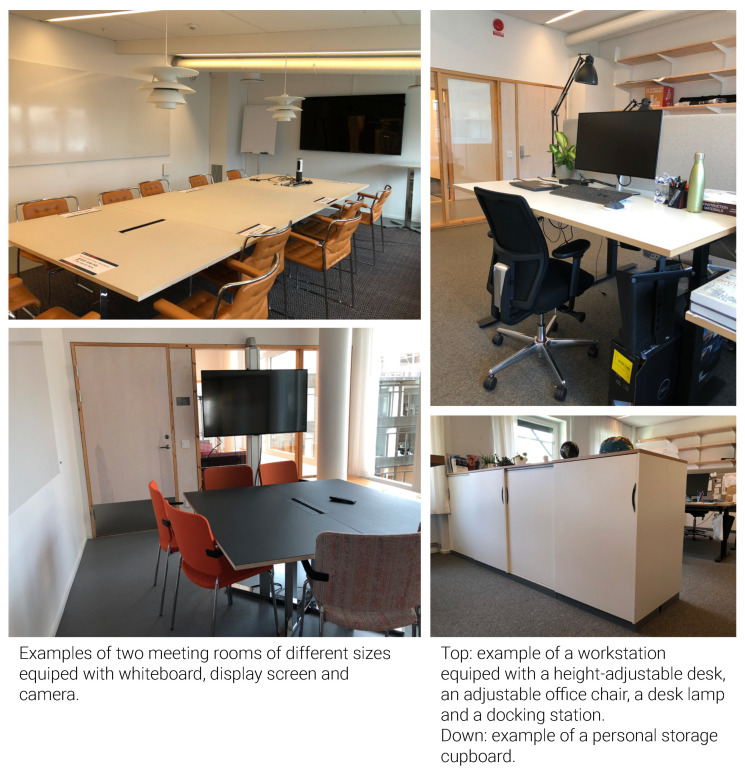
Access to resources.

Design setting: All employees had access to resources, including a uniformly sized personal storage cupboard, shelves, a height-adjustable desk, an adjustable office chair and a docking station in the office rooms. Meeting rooms were equipped either with or without a whiteboard, display screen, video projector and webcam. Flex rooms had cupboards with one screen per desk (Figure 9).

Perceptions: The majority of interviewees (31/41) found their needed resources to be available and accessible. High-quality and adjustable furniture, adequate storage space and good IT equipment were found to be important to manage one’s work. An appreciable minority (10/41) experienced a lack of storage space, difficulties working with equipment and a lack of IT support and training.

#### 4.2.3. Participation and Involvement

Participation and involvement refer to change processes facilitating or hindering building user involvement.

Design setting: In an interview, the change coordinator confirmed that staff participation in the design process was limited to choosing between some predefined solutions provided by the architects. The change process was communicated through a newsletter and organization internal platform.

Perceptions: While only a few interviewees (7/41) perceived the opportunity for participation in pre- and/or post-relocation change processes, 16/41 interviewees did not perceive the possibility. The interviewees found the opportunities more formal than practical in that the final solutions were predetermined and that employees’ input was not considered. Overall, the perceived limited involvement in the change processes reduced the sense of empowerment and manageability. This also reduced meaningfulness by creating a sense that the employees’ opinions were underappreciated.

#### 4.2.4. Life Management

Life management refers to amenities that facilitate or hinder employees in balancing the pressure of work life.

Design setting: The observations and floor plans showed that facilities such as a resting room, changing rooms and bicycle storage were provided. Accessible changing rooms with cupboards and showers were located in the basement, one for female staff and one for male staff, and the bicycle storage was located in the basement of the building next door. However, no bicycle rack was provided in the storage, decreasing the security of the room. The resting room was located on the 3rd floor with a solid curtain on both sides, being the only room in the building that offered complete isolation.

Perceptions: The majority of the interviewees (35/41) perceived high decision latitude for choosing where and when to work due to the organization’s trust-based culture. The freedom to be in control of one’s own work schedule helped manage work-life balance. Nearly a quarter of interviewees reported a lack of access to proper biking facilities such as showers, changing rooms, and enclosed bicycle storage. Only a minority of interviewees (5/41) reported using the resting room on the third floor for relaxation and stress recovery.

### 4.3. Meaningfulness

**Figure 10:**
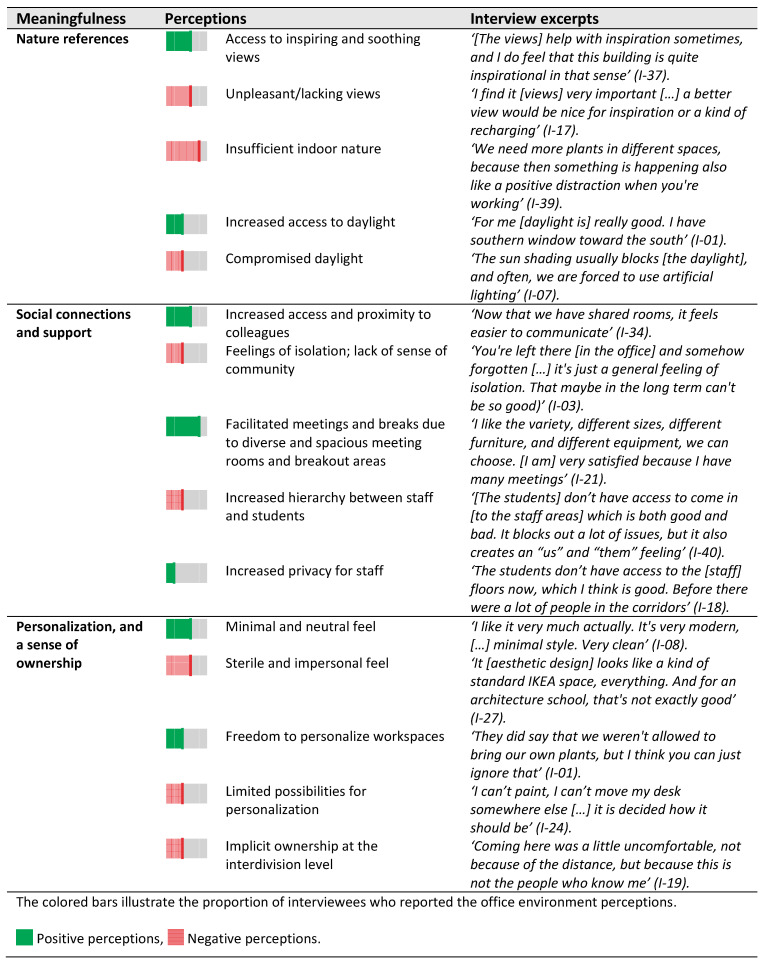
Perceptions of meaningfulness in the office environment as reported in interviews.

Meaningfulness in the office environment was associated with nature references, the social environment, personalization, and a sense of ownership (Figure 10).

#### 4.3.1. Nature References

Nature references refer to attributes that enable visual or physical access to the elements of the natural environment.

**Figure 11:**
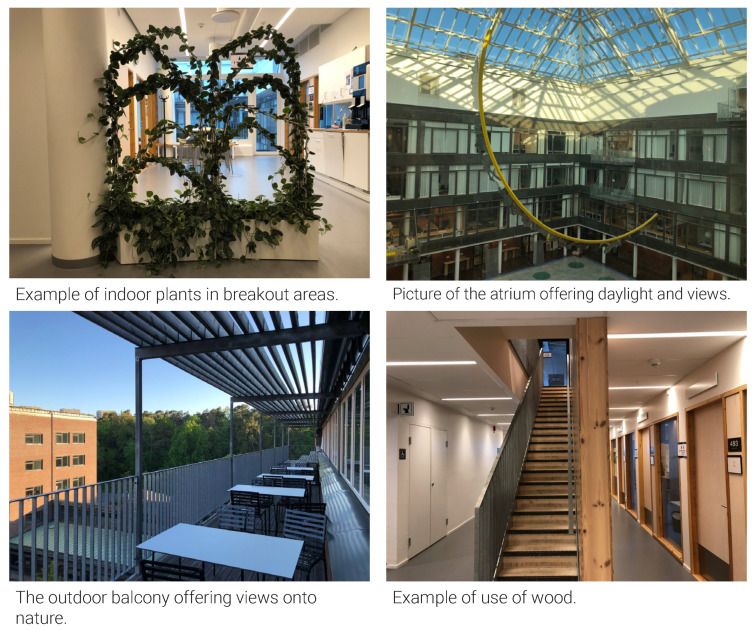
Nature references.

Design setting: All office rooms had windows with outdoor views. However, the northern and western façades faced the campus area and a hill, while the eastern façade faced a concrete wall, and parts of the northern façade faced a brick wall. The central atrium with a glass roof provided daylight and sky views, in addition to the interior balconies simulating an outdoor space (Figure 11). The southern exterior balcony in the lunchroom offered daylight and views onto the hill and trees. In terms of greenery, the same type of plants was placed in every breakout area. Another natural reference was wooden material used in the internal staircase and benches in meeting rooms.

Perceptions: Half of the interviewees (20/41) found the views from office windows and balconies inspiring and meaningful, while nearly the other half reported unpleasant views on a concrete and brick wall. Nature references such as greenery and plants in the office interior were perceived as insufficient by 26/41 interviewees. One-third of interviewees (13/41) appreciated the amount of daylight. However, the automated shades limited access to daylight for another third, making the office less meaningful.

#### 4.3.2. Social Connections and Support

Social connections and support refer to attributes facilitating or hindering social interactions and a sense of community in the office.

**Figure 12:**
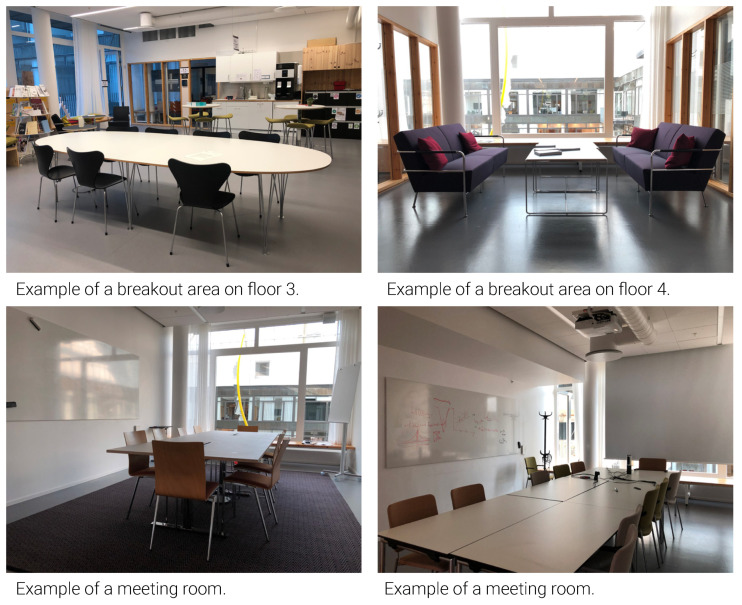
Social connections and support.

Design setting: The shared office rooms (2, 6 or 8 persons), as well as a high level of transparency between the office, back-up spaces and the corridor, increased visibility due to the use of glass partitions and thus increased access to colleagues. The two large breakout areas on the third floor accommodated larger groups of people than those on the fourth floor, which had a maximum of six persons (Figure 12). The lunchroom on the fifth floor had the largest capacity which was also the most often occupied room among back-up spaces. That said, none of the breakout areas were observed in use at their maximum capacity (Table 4). As students did not have access to staff areas (for security reasons), they could meet with teachers either in classrooms or in meeting rooms facing the staircases. The meeting rooms were furnished with chairs and tables. Soft furniture was provided only in breakout areas as well as two of the quiet rooms.

Perceptions: Over half of the interviewees (23/41) perceived increased access to colleagues and preferred face-to-face interactions for quick exchange of information. The majority of interviewees (36/41) also appreciated the diverse, proximate meeting rooms and breakout areas, which facilitated meetings and breaks with colleagues and hence improved meaningfulness. In particular, the balconies were among the most popular spaces in the breakout areas, offering a bright, relaxing environment for the majority of the interviewees (31/41). Conversely, 12/41 interviewees experienced a lack of sense of community due to difficulties in locating colleagues. This eventually led to feelings of isolation and thereby office environment was perceived less meaningfulness. Some interviewees (5/41) found limited student access to staff areas beneficial to managing one’s privacy. However, others (9/41) perceived a subsequent increase in the hierarchy between students and teachers.

#### 4.3.3. Personalization and Sense of Ownership

Personalization and aesthetics refer to attributes of the environment facilitating or hindering building users from adding personal/professional items to their work environments.

**Figure 13:**
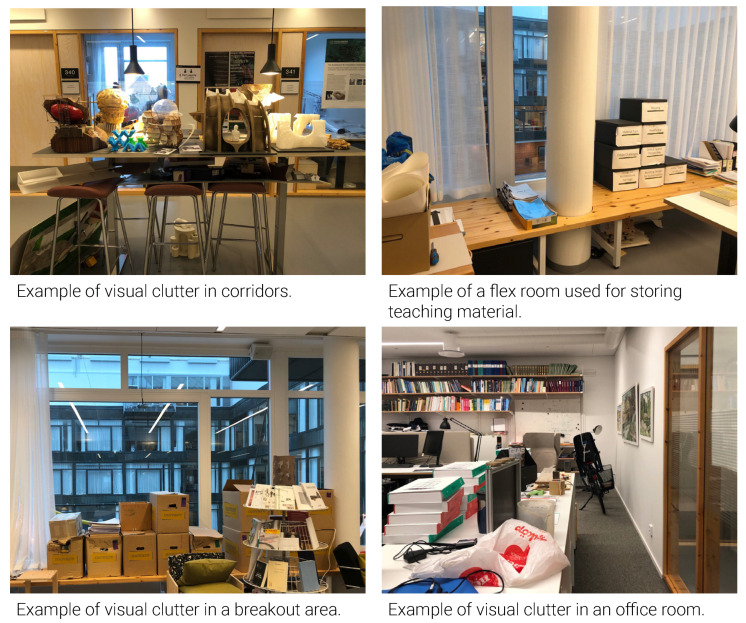
Visual clutter.

**Figure 14:**
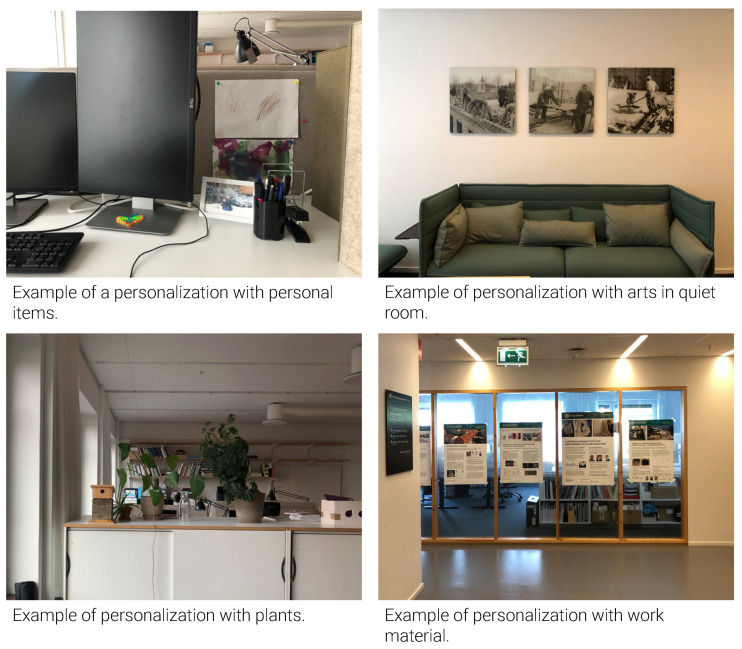
Examples of personalization.

Design setting: The colors used in the office interior were mostly neutral (white, gray, black) and earthy (wood, beige). Some parts of the office floors were cluttered with books, folders, models and moving boxes (Figure 13), often without clear indication of who ‘owned’ these items. The guidebook stated, ‘Enjoy the shared space, but please leave private furniture, textiles and plants at home’, thus discouraging employees from personalizing their workspace. Nevertheless, traces of identity expressions were found in office rooms, workstations, corridors, and back-up spaces, such as art, plants, photos, personal and professional items (Figure 14).

Perceptions: Almost half of the interviewees (20/41) appreciated the minimalistic and neutral look. Nevertheless, 16/41 found the aesthetic design of the office too ‘sterile’ and ‘impersonal’, which reduced office meaningfulness (Figure 10). Over one-third of interviewees (14/41) perceived the possibility of personalizing their office space, despite being aware of the discouragement by the organization. However, a few found the rules about personalization ambiguous and preferred to not add any personal items. Implicit ownership was signaled by groups of employees frequenting certain back-up spaces or breakout spaces and personalizing those spaces (e.g., with books, magazines, posters, models). The implied ownership was also reflected by 16/41 interviewees (16/41), indicating that they would feel uncomfortable using spaces on other ‘floors’ or ‘sides’ of the building to which they did not belong.

## 5. Discussion

This study investigated the current office design circumstances and employees’ perceptions of the office environment in relation to their SOC. The overall goal was to contribute to the understanding of interrelations between the office design, the perceived office environment and employees’ SOC. The findings about the office environment are discussed in relation to SOC. Additionally, methodological concerns and practical implications are addressed.

### 5.1. Office Environment in Relation to Sense of Coherence

The findings indicate that employees had a different conception of place than intended by the designer. That is, the potential of the office environment, such as biking facilities, was not perceived by the employees, reflecting low office comprehensibility. This could be due to a lack of communication with employees concerning the facilities provided. In contrast, the employees found potential in the office environment, which led to deviation from intended use. These acts have been conceptualized as the ‘misuse’ of architecture and as one of the reasons why flexible office concepts do not work as intended [22]. Babapour et al. [37] suggested that misuse may partly be a result of insufficient employee involvement in the design and planning processes as well as the use phase. From a design perspective, Søiland [19] argued that by attempting to repurpose workspaces to adapt them to their needs and preferences, employees negotiate their office design during use. In other words, users participate in design outside of organized user participation processes [19]. If the (use of the) office environment is seen as a product of users’ ongoing experience and understandings [38], there seems to be a disconnect between the experience and understandings of users and designers. This is in line with Canter’s theory on the psychology of place, in which he argued that our understanding of a place comes from our previous experiences that subsequently affect our behavior in it [23]. That is, through interactions with the office environment, employees develop and attach patterns of associations, expectations, and use [23]. Hence, employees’ spatial comprehension may influence the variety of meanings they assign to places and the ways in which they utilize workspaces (manageability).

The study shows that the components of SOC are interrelated. For instance, transparency and openness increased opportunities for interaction and were associated with positive meanings. However, this also led to difficulties in managing acoustic and visual distractions. This finding aligns with previous associations between open office environments and increased noise and lack of privacy [39]. Hence, more communication does not always improve employees’ SOC. Furthermore, feelings of isolation and a lack of sense of community among employees may have been due to the abundance of breakout areas, leading to a less meaningful office environment. A similar effect was found in activity-based offices where employees had difficulties locating colleagues at the office, which eventually had a negative impact on the sense of community and team cohesion in the long term [40,41]. Relatively few connections have been made between the office environment and social well-being [24,42]. This calls for a more detailed analysis of interrelations between inter/intrateam communications and architectural design features of social areas to be able to support employees in managing their meaningful social relations and exposure to stimuli.

Another interrelation is exemplified by personalization and subsequent feelings of confusion for employees. While employees were discouraged from adding personal items to the environment to assert meaning, some found the rules ambiguous (comprehensibility). Nevertheless, personalization of space is used as a means of making sense of the environment and giving meaning to the workspace [43,44].

The study highlights the relation between the (use of the) office environment and facility management in terms of behavioral rules, lack of training, and a maintenance system experienced as inconsistent with the follow-ups. The importance of clear behavioral rules for successful implementations [22,45] as well as employee involvement in the change process have been emphasized by previous research [16,46,47,48]. Research has highlighted the importance of incorporating ergonomic training when introducing new office design to optimize the experience of flexible offices [49]. Furthermore, the frustration caused by the maintenance system is consistent with other studies showing that a sense of resignation occurs when management does not address issues that disrupt employees’ work [50]. The study thus underlines the crucial role of facility management in creating comprehensible, manageable, and meaningful office environments through engaging in recurring communication and dialog with employees.

### 5.2. Methodological Concerns

The adopted qualitative case study approach fits the contextual nature of architectural design and health [33] and is considered useful for studying individuals or groups within their specific context [51]. Although the case study approach is criticized for its dependency on a single case exploration, it has been argued that the parameter and goal setting of the research–in this case the SOC framework– are more important than a large sample size [51]. This study highlighted the interrelations between the office environment and employees’ SOC using the specificity of the studied office design rather than establishing cause-effect relationships between variables. The findings cannot therefore be generalized to other cases. Our study findings are instead transferable, as the study concerns experiences of SOC in an office environment, which are diverse for different employees and expectedly so for different cases. According to the criteria for ensuring the quality of qualitative studies [52], reliability was ensured through a thorough and transparent description of the case, triangulation of multiple data sources, and ongoing discussions between the researchers to ensure a consistent analysis strategy. Future research may benefit from our findings in developing survey instruments to assess SOC in the office environment and provide more generalizable insights.

A key strength of the study approach was its objective design perspective in combination with the perceptions of employees. Given the variety of layouts, sizes, implementation processes, etc., flexible offices should be studied with attention to their design differences in order to capture diverse experiences. The study provided insight on the case-specific design aspect which contributes to the development of a sound evidence base. This enables more accurate comparisons between different cases and helps to map positive and negative design aspects in flexible office environments. The study also provides in-depth insights regarding a range of aspects, from risk factors for health (pathogenic aspects) to health-promoting potential of office environments (salutogenic aspects), in response to previous calls for positive approaches toward health) [1,2,3,4]. Investigations on what makes an office design healthy or about the interrelations between office environments and employees’ health may benefit from adopting such case study approaches.

Notably, the involvement of design professionals as participants may have resulted in negative bias since design professionals might be more critical about space [53]. However, they may also be more conscious of their office environment and therefore may be able to provide more detailed insights.

### 5.3. Practical Implications

From a salutogenic perspective, previous research has shown that when people are healthy, they demonstrate a theoretical surplus of coping resources [54]. Nevertheless, when people are ill, they struggle in the balance between deterioration and recovery [54]. So, for people who have poorer health conditions, it becomes even more important to provide a healthy office wherein the features of the environment help individuals cope with work and everyday life.

This study focused on the salutogenic factors of the office environment outlined in the SOC framework. The findings showed that when these factors fit employees’ needs and preferences, they become resources to cope with challenging conditions. Nevertheless, the same factors can become deficits, when they are suboptimal, hindering employees’ SOC.

The negative perceptions regarding various aspects of the office environment highlight suboptimal design features with regard to employees’ SOC. The following modifications are based on authors’ interpretations and concern continual enhancements in the office environment that may improve SOC through, for instance, the introduction and communication of expected behaviors, the addition of meaningful items to the office environment, and increased control by adding space-level control over stimuli (Table 5).

## 6. Conclusions

The findings showed that not all potential of the office environment was perceived by the employees. Additionally, the ways that employees appropriated the office environment were not planned by the designers. The study also highlighted interrelations between the SOC components. Low levels of office comprehensibility caused by a lack of behavioral rules, information about existing facilities, and facility management/maintenance subsequently led to reduced manageability and meaningfulness. Discouragement of personalization and a subsequent feeling of confusion for employees limited comprehensibility and meaningfulness. Moreover, the facilitated access to colleagues, which was perceived as meaningful, led to more distractions and reduced manageability. The study noted that spatial transparency and openness did not prevent feelings of isolation among some employees and that more interaction is not always better for SOC. This finding implies that organizations may need to prioritize which components of SOC should be supported most by the office environment. Furthermore, it suggests that case-specific design aspects should play a more central role in studying and conceptualizing healthy office design and that design solutions should be continuously modified during the use phase, while ensuring employees’ participation.

As outlined in the introduction, a question of interest to organizations and practitioners is how office environments should be planned, designed, and managed to support or enhance health. This question cannot be suitably addressed when the specificity of design is overlooked. Furthermore, the study notes that an ‘ideal’ office environment should not be the goal. Instead, office design should provide an environment in which employees are able to cope with challenges in comprehensible, manageable and meaningful ways.

## Figures and Tables

**Table 1:** The participants’ demographics and professions.

Demographics	Total Invited (*n* = 238)	Participants (*n* = 41)
	Female = 109 Male = 129	Female = 19 Male = 22
Researcher, professor, lecturer	192	29
Project assistant/guest researcher	13	4
Other categories (e.g., project manager, admin)	33	8
Interviewee’s time working in the organization	0–1 yrs. = 21.9% 2–5 yrs. = 41.4% >6 yrs. = 36.5%	
Interviewee’s age range	24–30 = 29.2% 31–40 = 29.2% 41–50 = 19.5% 50 ≥ 21.9%	

**Table 2:** Sample questions from the interviews in relation to SOC.

Sense of Coherence	Interview Questions
Comprehensibility	Are there any rules or agreements between colleagues on how to use the different office zones depending on your activity? (If yes) Are those rules respected? (If no) Do you wish to have them?
Manageability	What do you do when your work demands high concentration? Where do you concentrate? How? Why? How do you approach people when you need to ask/tell something to someone?
Meaningfulness	How do you socialize with your colleagues at the office?

**Table 3:** Interview coding strategy.

Excerpt	Step 1 Perceptions of Office the Environment	Step 2 Office Environment Features	Step 3 Sense of Coherence Components
‘*It’s supposed to be a quiet room, but it isn’t. So, people tend to sit here and discuss matters and prepare’* (I-15).	Use of quiet rooms for spontaneous/informal discussions and phone calls	Understanding the function of space	Comprehensibility
*‘I’m a very anxious person, so it takes me out of my zone to feel observed’* (I-14).	Exposure to visual stimuli	Control over the environment	Manageability
*‘Now that we have shared rooms, it feels easier to communicate’* (I-34).	Increased access and proximity to colleagues	Social connections and support	Meaningfulness

**Table 4:** Occupancy during a working week.

Avg. Occupancy *	**(%)**
Office rooms, 2 persons	25.9
Office rooms, 6–8 persons	28.29
Avg. Utilization **	
Meeting rooms, 4–6 persons	27.4
Meeting rooms, 6+ persons	28.2
Quiet room with sofa	11.1
Quiet rooms, 2 persons	30.5
Quiet rooms, 6 persons	44.4
Flex rooms	69.4
Phone booths	14.5
Breakout areas	22.2
Lunchroom-5th floor	88.8

* Percentage of workstations occupied with respect to maximum number of workstations. ** Percentage of the total number of 18 observations that the spaces were observed in use.

**Table 5:** Proposed modifications.

Suboptimal Comprehensibility Features	Design Setting	Proposed Modifications
Wayfinding		
Difficulties orienting in the building	Symmetrical layout Repetitive furniture; lack of visual clues Square layout	Add a distinct labeling system; add visual clues, e.g., assign a color per staircase, add distinct furniture particular to a corner, provide ‘you are here’ maps.
Understanding the functions		
Quiet rooms used for spontaneous/informal discussions	Not reservable Soft and facing furniture	Allocate meeting rooms with soft furniture for informal meetings. Implement a booking system. Take out large couches from quiet rooms and replace with armchairs that offer visual seclusion.
Phone booths for concentrated work	Low transparency level; minimal distractions Signaling of unavailability	Dedicate enclosed spaces with visual protection for concentrated work. Use signage to communicate behavioral rules.
Meeting rooms for individual, and concentrated work	Availability Whiteboard and large table	Introduce multipurpose rooms for individuals and project teams. Provide large meeting tables that allow for laptop use, note taking or discussing large drawings
Reception area perceived as an undefined space	No receptionist Lack of information for visitors	Clearly communicate the function of the reception area with physical and digital information boards. Showcase the research and education carried out by the department to those from outside the department.
Meeting rooms with 4-person capacity perceived too small for 4 persons	Small table for four laptops Small area per employee (2.5 m2/person)	Reduce the room capacity to 2 persons. Include large meeting tables that allow for laptop use, note taking or discussing large drawings.
Behavioral rules		
Exposure to conversations and noise	Ambiguous office etiquette	Use signs on workstations to communicate availability, e.g., ‘Do not disturb’ or ‘I am available’.
Mess and visual clutter	Provided in-house guidebook	Increase enclosed storage space for teaching, research, and administrative materials, e.g., by implementing a modular storage cabinet. Communicate a clear protocol and office etiquette regarding extra equipment, work materials, cleaning, and hygiene of common spaces. Follow up to ensure the office etiquettes are complied with. Formally dedicate breakout areas to groups and direct responsibilities for maintenance and cleanliness.
Information sharing and transparency		
Ambiguous maintenance procedure	Provided phone numbers for maintenance at doors No follow-up system	Implement a responsive maintenance system e.g., by assigning a follow up number to each problem report.
**Suboptimal manageability features**	**Design setting**	**Proposed modifications**
Control over the environment		
Disturbance due to low temperature	Centralized climate system and lack of control	Raise the temperature. Provide extra heaters.
Disturbance by automated shades	Malfunction of automated shades and lack of control	Enable manual control over daylight e.g., with opaque curtains.
Exposure to visual stimuli Exposure to acoustic stimuli	Glass partition and high level of transparency Shared office rooms Lack of behavioral rules	Provide opaque curtains and dividing panels between workstations. Use signs on workstations to communicate availability, e.g., ‘Do not disturb’ or ‘I am available’. Provide a range of solutions from noise-cancelling headphones to sound-absorbing panels and quiet rooms.
Poor soundproofing	No sound insulation provided in meeting rooms or phone rooms	Improve Soundproofing of meeting rooms e.g., by adding sound-absorbing panels.
Access to resources		
Difficulties working with equipment and ergonomics	No training provided	Provide digital and physical training and instructions on how to set up and use technical equipment.
Limited storage space	All employees with assigned desks have access to a storage cupboard	Increase enclosed storage space for teaching, research, and administrative materials, e.g., by implementing a modular storage cabinet.
Participation and involvement		
Limited possibilities for involvement in pre- and/or post-relocation processes	Limited employee involvement in change processes	Implement yearly workshops to discuss the physical work environment and ensure employee involvement in the change processes.
Life management		
Lack of bike facilities	Changing room, bicycle storage, and bicycle parking are provided.	Introduce and communicate about the facilities with the employees, make them accessible, and provide secluded bike storage with locks.
**Suboptimal meaningfulness features**	**Design setting**	**Proposed modifications**
Nature references		
Unpleasant/lacking views	Views onto brick and concrete walls (east-facing and part of north-facing façades)	Investigate the possibility to allocate unpleasant façade sides to short term activities such as phone rooms, or video recording rooms.
Insufficient indoor plants and greenery	Similar plants in breakout areas	Add more plants and allow employees to choose the type of plants. Encourage employees to bring their own plants.
Compromised daylight	Automated shades limiting access to direct daylight	Enable manual control over daylight e.g., through opaque curtains
Social connections and support		
Isolation and lack of sense of community	Low capacity and/or abundance of breakout areas; lack of allocated breakout area for divisions	Allocate breakout areas to different groups. Organize collective activities for personalization of breakout areas. Implement a break schedule in allocated breakout areas.
Increased hierarchy between teachers and students	Lack of student access to teachers’ area	Furnish the reception area as a break area where staff and students can informally meet.
Personalization and sense of ownership		
Sterile and impersonal feel	Use of neutral and earthy colors; discouragement from personalizing workspaces	Add art and posters (e.g., nature, design/engineering related photos) to the meeting rooms to underline the type of university building.
Limited possibilities for personalization	Discouragement from personalizing workspaces	Allow personalization in offices with some guidelines in terms of clutter.
Implicit ownership at the intradivision level	Proximity to workstations Signaling of ownership through personalization and frequenting of specific spaces	Formally dedicate breakout areas to groups and direct responsibilities for maintenance and cleanliness.

## Data Availability

The full data are not publicly available due to ethical/privacy reasons.
